# An Efficient Genetic Transformation and CRISPR/Cas9-Based Genome Editing System for Moso Bamboo (*Phyllostachys edulis*)

**DOI:** 10.3389/fpls.2022.822022

**Published:** 2022-02-11

**Authors:** Biyun Huang, Renying Zhuo, Huijin Fan, Yujun Wang, Jing Xu, Kangming Jin, Guirong Qiao

**Affiliations:** ^1^State Key Laboratory of Tree Genetics and Breeding, Chinese Academy of Forestry, Beijing, China; ^2^Key Laboratory of Tree Breeding of Zhejiang Province, Research Institute of Subtropical of Forestry, Chinese Academy of Forestry, Hangzhou, China

**Keywords:** *Phyllostachys edulis*, plant regeneration, genetic transformation, genome editing, immature embryo culture

## Abstract

Moso bamboo (*Phyllostachys edulis*) is the most important monopodial bamboo species worldwide. Without a genetic transformation system, it is difficult to verify the functions of genes controlling important traits and conduct molecular breeding in moso bamboo. Here, we established a plant regeneration system from immature embryos. Calli were induced on MS medium added 4–6 mg⋅L^–1^ 2,4-dichlorophenoxyacetic acid (2,4-D) with high efficiency (>60%). A plant growth regulator combination of 0.5 mg⋅L^–1^ 1-naphthylacetic acid (NAA), 2.0 mg⋅L^–1^ 6-benzylaminopurine (BAP), and 3.0 mg⋅L^–1^ zeatin (ZT) was suitable for shoot differentiation, and the shoot induction frequency was increased to 43% after 0.5 mg⋅L^–1^ abscisic acid (ABA) pretreatment. An effective antibiotic screening concentration was determined by hygromycin sensitivity test. We further optimized the *Agrobacterium* concentration and added vacuum infiltration for infection, which improves the transient expression efficiency. A genetic transformation system was established for the first time in moso bamboo, with the transformation efficiency of approximately 5%. To optimize genome editing, two endogenous U3 small nuclear RNA (snRNA) promoters were isolated and used to drive small guide RNA (sgRNA) expression. The results showed that the *PeU3.1* promoter exhibited higher efficiency, and it was used for subsequent genome editing. Finally, homozygous *pds1pds2* mutants were obtained by an efficient CRISPR/Cas9 genome-editing system. These technical systems will be conducive to gene functional validation and accelerate the molecular breeding process of moso bamboo.

## Introduction

Bamboo is an important forestry resource with a global bamboo forest area of more than 47 million hm^2^ and an annual output of more than 500 million tons of bamboo wood (Li and Fu, 2021). Moso bamboo (*Phyllostachys edulis*) is the most economically important woody bamboo species and plays essential economic and ecological roles. This species is characterized by asexual reproduction, rapid growth, and a high carbon fixation capacity (Zhou et al., 2011). Recently, an analysis of 427 genomes has revealed the low genetic diversity of the moso bamboo population, which indicates that this species may have a low effective population size and a small genetic pool that can be used for future breeding purposes (Zhao et al., 2021). Unfortunately, due to the long and unpredictable flowering cycle of bamboo, it is difficult to obtain new varieties through crossbreeding. Therefore, identifying the genes controlling important bamboo traits and using molecular breeding to improve varieties are the methods that can be used to obtain new varieties quickly.

The genome of moso bamboo has been released (Peng et al., 2013) and assembled at the chromosome level (Zhao et al., 2018), which enables major advances in understanding bamboo plants. To date, the mechanisms of critical traits that include flower formation, rapid growth, shoot primary thickening growth, and lignification in moso bamboo have mainly revealed by omics (Ge et al., 2017; Wei et al., 2017; Wang et al., 2021; Yang et al., 2021). Numerous candidate genes have been identified, but there has been no functional verification of any gene in moso bamboo due to the lack of a genetic transformation system. Plant regeneration systems of bamboo have mainly been reported in sympodial bamboo species. Since the first report of plant regeneration from the zygotic embryos of *Bambusa arundinacea* (Mehta et al., 1982), plant regeneration protocols have been conducted in several sympodial bamboo species from mature embryos of *Dendrocalamus latiflorus*, *Dendrocalamus strictus*, and *Otatea acuminata* aztecorum (Rao et al., 1985; Yeh and Chang, 1987; Woods et al., 1992), or from anthers of *Bambusa oldhamii*, *Bambusa beecheyana var. beecheyana*, and *D. latiflorus* (Yeh and Chang, 1986a,b; Tsay et al., 1990; Qiao et al., 2013). Recently, plantlets successfully regenerated from shoot tips of *D. hamiltonii* and young shoots of *D. latiflorus* were reported (Zang et al., 2016; Ye et al., 2017). There has been only one reported case of monopodial bamboo species, which shows plant regeneration from calli induced from mature seeds of *P. edulis* (Yuan et al., 2013). However, the mature seeds were difficult to be completely sterilized, and no more than 5.0% of the calli were able to regenerate. A genetic transformation system has been established only in Ma bamboo (*Dendrocalamus latiflorus*), which represents sympodial bamboo species (Qiao et al., 2014; Ye et al., 2017). Moso bamboo is a monopodial bamboo species, which has different growth traits from sympodial bamboo.

Genome editing is a new genetic engineering technology that can accurately modify the specific target sites on the genome. It mainly includes artificial nuclease-mediated zinc finger nucleases (ZFNs), transcription activator-like effector nucleases (TALENs), and RNA-guided CRISPR/Cas nucleases (CRISPRs) (Bibikova et al., 2003; Bedell et al., 2012; Cong et al., 2013). Genome editing shows great potential in basic research and crop genetic improvement. Third-generation genome-editing technology, such as CRISPR/Cas9, has incomparable advantages over other genome-editing technologies. Cas9 protein combined with single-guide RNA can not only play the role of recognition, but also perform cutting function and target-specific sites. The CRISPR/Cas9 system, as an efficient gene site-specific editing technology, has been successfully applied to *Arabidopsis thaliana*, rice (*Oryza sativa*), maize (*Zea mays*), wheat (*Triticum aestivum*), and poplar (*Populus davidiana* × *Populus bolleana*) (Zhang et al., 2016, 2018; Liang et al., 2017; Lee et al., 2018; Papikian et al., 2019). At present, the realization of genome editing mainly depends on plant regeneration and *Agrobacterium*-mediated transformation. An efficient regeneration and genetic transformation system is the premise of efficient genome editing. However, there has only been one report on the application of gene editing in bamboo. In that report, a *dlmpsy1* mutant with albino phenotype and a mutant with altered plant height were generated by CRISPR/Cas9 (Ye et al., 2020).

Promoters of *U3*/*U6* small nuclear RNA (snRNA) with transcriptional initiation sites A/G are important for driving small guide RNA (sgRNA) transcription in CRISPR/Cas9 system. *U3* or *U6* promoters with high transcriptional activity can accurately guide the transcription of sgRNA, so as to reduce the off-target effect caused by unrelated DNA transcription (Li et al., 2007; Belhaj et al., 2013; Cong et al., 2013). Both endogenous and exogenous *U3*/*U6* promoters have been successfully applied to drive sgRNA expression in different plants. However, it has been reported that species-specific *U3*/*U6* promoters are much more efficient for driving sgRNA transcription and enhancing the editing efficiency of the CRISRP/Cas9 system (Feng et al., 2013; Sun et al., 2015; Long et al., 2018). In cotton, the genome-editing efficiency increased 4–6 times when sgRNA was driven under the endogenous *GhU6.3* promoter compared with exogenous *AtU6-29* promoter (Long et al., 2018). The transcription efficiency was also different between *U3*/*U6* promoters of the same species. The editing efficiencies of five endogenous *U6* promoters were different and varied from 8.47 to 24.92% in *Hevea brasiliensis* (Dai et al., 2021). Therefore, identifying additional endogenous *U3*/*U6* promoters is conducive to the improvement in the CRISPR/Cas9 genome-editing system. In this study, we first established an efficient plant regeneration system from immature embryos and a genetic transformation system *via Agrobacterium tumefaciens* in moso bamboo. The factors that affect plant regeneration and transformation efficiency were optimized. Two endogenous U3 snRNA promoters were identified from moso bamboo for driving sgRNA expression, and the editing efficiency was compared to *OsU3* promoter. Finally, taking the editing of the *PePDS* gene as an example, the applicability of an efficient CRISPR/Cas9-based genome-editing system was first demonstrated in moso bamboo. These technical systems will be conducive to gene functional validation and accelerate the molecular breeding process in moso bamboo.

## Materials and Methods

### Plant Materials

**FIGURE 1:**
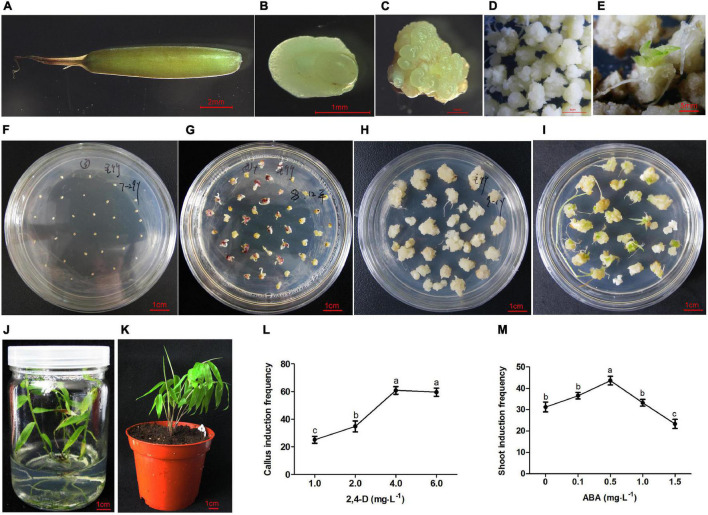
Plant regeneration from immature embryo of moso bamboo. **(A)** Immature seed. **(B)** Immature embryo. **(C)** Callus inducted from embryo. **(D)** Callus proliferation. **(E)** Plantlet differentiated immediately on the subculture medium. **(F)** Immature embryos cultured on the induction medium. **(G)** Calli inducted from embryos on the induction medium for 3 weeks. **(H)** Calli proliferation on the subculture medium. **(I)** Shoots induction on the differentiation medium. **(J)** Rooting and plant regeneration. **(K)** Regenerated plant was transplanted into soil. **(L)** Callus induction frequency on MS medium containing 2,4-D 1–6 mg⋅L^–1^. **(M)** Shoot induction frequency pretreated by ABA at different concentration of 0–1.5 mg⋅L^–1^. Different lowercase letters showed significant difference by Tukey’s multiple comparison test, *p* < 0.05.

Immature seeds were collected from a natural flowering moso bamboo forest (25°12′36″*E –* 110°46′12″*N*) in Guangxi Zhuang Autonomous Region, China, from July to August. The seeds, which were plump, grayish-green (Figure 1A), were washed under tap water for 2 h and then soaked in 75% ethanol for 1 min. After being sterilized in a 2% sodium hypochlorite solution containing 0.1% Tween-80 for 15–20 min and rinsed with autoclaved distilled water 3–5 times, the embryos (about 1.3 mm in diameter) were extruded from the base of the seeds with a sickle probe on sterile filter paper and placed onto individual 9-cm diameter petri dishes containing 20-ml induction medium (Supplementary Video 1). The calli were induced and proliferated in the dark, whereas shoot differentiation and rooting were conducted at 25°C under a 16 h light/8 h dark photoperiod with a light intensity of 60–70 μmol/m^2^/s. For callus and shoot induction frequency, three repetitions were set up with 100 explants or calli per replicate. The statistical analysis was analyzed using SPSS software version 22.

### Callus Induction and Plant Regeneration From Immature Moso Bamboo Embryos

For callus induction, different concentrations of 2,4-dichlorophenoxyacetic acid (2,4-D) (1, 2, 4, and 6 mg⋅L^–1^) were supplemented to MS medium (MS basal medium with 30 g⋅L^–1^ sucrose and 8 mg⋅L^–1^ agar, pH 5.8). After 3 weeks of induction, light yellow calli were subcultured on MS medium with 0.1–1.0 mg⋅L^–1^ 2, 4-D once every month. Plant growth regulator combinations with different concentrations (0.1, 0.5, and 1.0 mg⋅L^–1^ 1-naphthylacetic acid, NAA, 1, 2, and 3 mg⋅L^–1^ 6-benzylaminopurine, BAP, 1, 3, and 5 mg⋅L^–1^ zeatin, ZT) were used for shoot induction. To further improve the differentiation efficiency, the calli were pretreated with 0, 1, 0.5, and 1.0 mg⋅L^–1^ abscisic acid (ABA) in subculture for 1 month and then transferred to shoot induction medium. For root induction, 1/2 MS basal medium supplemented with 2 mg⋅L^–1^ 3-indolebutyric acid (IBA) was used. After induction for 1.5 months, the regenerated plants with 5–7 cm height were transplanted into peat soil and cultivated in a growth chamber at 26°C with a 16-h light/8-h dark photoperiod.

### Genetic Transformation Mediated by *A. tumefaciens*

**FIGURE 2:**
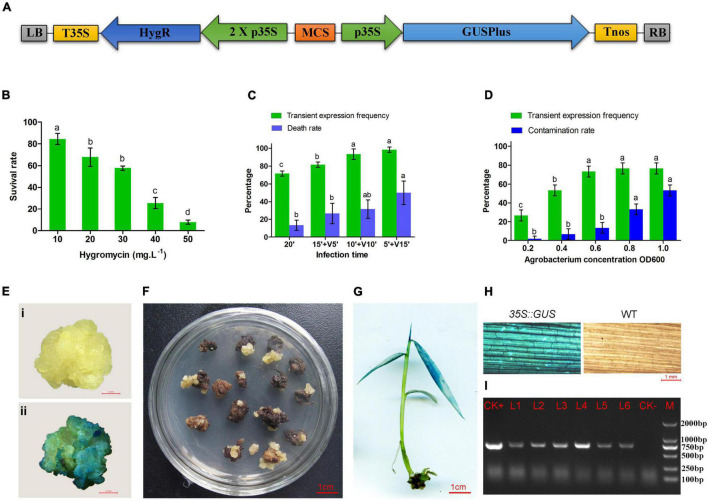
*Agrobacterium*-mediated transformation of moso bamboo. **(A)** Schematic representation of key elements of T-DNA in the binary vector pCAMBIA1305. LB, left border; T35S, terminator of CaMV35S; HygR, hygromycin phosphotransferase gene; 2 X p35S, CaMV 35S promoter (enhanced); MCS, multiple cloning sites; p35S, promoter of CaMV35S; GUSPlus,β-glucuronidase gene; Tnos, NOS terminator; RB, right border. **(B)** Hygromycin sensitivity of calli of moso bamboo. **(C)** Transient expression frequency and contamination rate of *Agrobacterium* at different concentration of *Agrobacterium*. **(D)** Transient expression frequency and death rate by vacuum infiltration for 5–15 min. Three repetitions were set up with 30 calli per replicate. Different letters on the same series showed significant difference by Tukey’s multiple comparison test, *p* < 0.05. **(E)** GUS staining of negative **(i)** and positive **(ii)** callus after cocultured 3 days. **(F)** Transformed callus on selected medium. **(G)** Positive plant detected by GUS staining. **(H)** Leaves of transgenic plant and wild type detected by GUS staining. **(I)** PCR detection of transgenic plants. M, DL2000 marker; CK+, plasmid; CK-, non-transgenic plant; L1–L6, transgenic plants.

To test the sensitivity of calli to hygromycin, the calli were cultured on the subculture medium with different concentrations of hygromycin (0, 10, 20, 30, 40, and 50 mg⋅L^–1^) for 4 weeks. Then, the calli were subcultured on a new medium containing hygromycin for 4 weeks. The callus survival rate was calculated by the ratio of the number of calli that proliferated to the total number of tested calli. In this study, *pCAMBIA1305* binary vector containing a *35:GUS* expression frame and a plant selectable marker gene *HygR* was transformed into *A. tumefaciens* strain *EHA105* (Figure 2A). Large white calli were infected in *Agrobacterium* suspensions with concentrations of OD600 0.2, 0.4, 0.6, 0.8, and 1.0 for 20 min. Vacuum infiltration for 5–15 min was performed at OD600 0.6, and the total infection time was 20 min. After 3 days of coculture on the subculture medium with 100 μmol⋅L^–1^ acetosyringone (AS), some of the transformed calli were used to test the transient expression frequency through β-glucuronidase (GUS) staining. The remaining calli were transferred to the selected medium with 40 mg⋅L^–1^ hygromycin and 300 mg⋅L^–1^ cefotaxime. After subculture and successive screening for 3–4 months, resistant calli were transferred to the shoot induction medium with 25 mg⋅L^–1^ hygromycin and 300 mg⋅L^–1^ cefotaxime. After 2–3 months, the hygromycin-resistant shoots were rooted on root induction medium with 300 mg⋅L^–1^ cefotaxime. The regenerated plantlets were transferred to a greenhouse and tested by GUS staining and PCR (primers: 35S-F: 5′-GACGCACAATCCCACTATCC; GUS-R: 5′-GTTACGAATGACTTTTCCGAGG; 785 bp). The transformation protocol has been provided in the Supplementary Material.

### Identification of PeU3 Promoter and Used for Small Guide RNA Expression

Two endogenous U3 snRNA promoters were identified from the moso bamboo genome. The sgRNA sites targeting PH02Gene09948 and PH02Gene04757 were designed by http://skl.scau.edu.cn/targetdesign/ and constructed under the *OsU3/PeU3.1/PeU3.2* promoter. The expression frame of *U3:sgRNA* was then introduced to *pC1300-Ubi:Cas9* vector according to previously reported methods (Wang et al., 2015). The constructs were transformed to *A. tumefaciens* strain EHA105, and then, the calli were infected. DNA was extracted the from the resistant calli and amplified by PCR (primers: Cas9-F: 5′-GGCATAAGCCCGAGAACAT, Cas9-R: 5′-CAAAAGTTGCCTCCAGTAGTTC, 404 bp). The positive samples were sequenced using the Hi-TOM protocol (Liu et al., 2019).

### CRISPR/Cas9 Genome Editing

To obtain stable transformed plants edited by CRISPR/Cas9, the homologous sequences of *OsPDS* were identified by BLAST searching the BambooGDB^1^. PCR primers were designed on the conserved regions of these two homologous sequences. The PCR products were sequenced by the Hi-TOM protocol to confirm the subgenomes. Conserved sgRNA sites in all copies were designed and driven by *PeU3.1* promoter and then introduced into *pC1300-Ubi:Cas9*. Transgenic plants were obtained through the transformation process described above and tested by PCR amplification. The positive plants were sequenced using the Hi-TOM protocol. The primers used included PDS - F: CAAGCACTGAAAAGTAGTCACCGT and PDS - R: AATCCTAGAATTACATCAGACC (331 bp).

### Phenotypic Observation by Transmission Electron Microscope

The leaves of albino mutants and wild types were cut into pieces less than 1 mm^2^ and fixed by 2.5% glutaraldehyde and 1% osmic acid. After dehydration with ethanol and acetone, the samples were embedded and cured. The tissues were then located using a semithin slicer (1 um) and sliced by an ultrathin slicer (70 nm). The sections were stained with 3% uranium acetate-lead citrate and observed by transmission electron microscope (JEOL l1230).

## Results

### Plant Regeneration From Immature Moso Bamboo Embryos

The immature embryos (Figure 1B) were separated from the seeds (Figure 1A) and cultured in the callus induction medium (Figure 1F). Preliminary experiments showed that the concentration of 2,4-D mainly affected the callus induction frequency. The embryos could germinate on the medium without any plant hormones, whereas germination was inhibited with the increase of 2,4-D concentration. The immature embryos remained in the induction medium for 10 days, where they began to swell and turn light yellow. Light yellow calli were induced on the MS medium with the addition of 1–6 mg⋅L^–1^ 2,4-D for 3 weeks (Figures 1C,G). The highest induction frequency was around 60% when 4 mg⋅L^–1^ 2,4-D was added to the MS medium. However, there was no significant change when the concentration was increased above 4 mg⋅L^–1^ (Figure 1L).

Light yellow calluses were subcultured on MS medium with low concentrations of 2, 4-D (0.1–1.0 mg⋅L^–1^) once every month. During this period, calluses in different states including yellow compact, white block, and white globular calli were produced. These calli could proliferate in large numbers (Figures 1D,H), and some of them (5%) could differentiate into plantlets immediately during subculture (Figure 1E). Various combinations of cytokinins and auxins were tested at different concentrations (Table 1). The frequency of shoot induction was about 34.5% on MS medium supplemented with 2.0 mg⋅L^–1^ BAP, 3.0 mg⋅L^–1^ ZT and 0.5 mg⋅L^–1^ NAA (Figure 1I). To further improve the shoot induction frequency, calli were pretreated with different concentrations of ABA and showed higher differentiation rate of 43% at 0.5 mg⋅L^–1^ ABA pretreatment (Figure 1M).

**TABLE 1:** Effect of plant growth regulator on shoot induction of moso bamboo.

No. PGR	BAP	ZT	NAA	Shoot induction frequency
1	1	1	0.1	16.75 ± 2.03^de^
2	1	3	1	18.45 ± 2.00^de^
3	1	5	0.5	24.58 ± 1.13^bc^
4	2	1	1	15.19 ± 1.37^e^
5	2	3	0.5	34.5 ± 1.17^a^
6	2	5	0.1	21.19 ± 1.95^cd^
7	3	1	0.5	20.95 ± 2.63^cd^
8	3	3	0.1	28.65 ± 2.13^b^
9	3	5	1	20.17 ± 1.33^cde^

*Different lowercase letters show significant difference by Tukey’s multiple comparison test, p < 0.05.*

When the shoots grew to more than 1.5 cm, they were transferred to the rooting medium. The roots could be inducted on 1/2 MS basal medium supplemented with 2 mg⋅L^–1^ IBA. The rooting rate was about 95% at 3 weeks after induction (Figure 1J). The regenerated plants were transplanted into peat soil, and the survival rate was close to 100% (Figure 1K).

### Genetic Transformation Mediated by *A. tumefaciens*

Large white calli were selected as the receptors for gene transformation. First, different concentrations of hygromycin (0–50 mg⋅L^–1^) were set up for optimization of the concentration of antibiotic for transformation selection. Some calli turned brown after culture on the medium containing hygromycin for 4 weeks. The browning calli could not grow and proliferate, whereas some slightly browning or non-browning calli could still grow new calli after being subcultured on the same medium for another 4 weeks. The survival ratio of calli decreased significantly with increased concentrations of hygromycin (Figure 2B). Finally, 40 mg⋅L^–1^ hygromycin was used to screen the putative transgenic plants.

According to the traditional transformation method, the calluses were infected in the bacterial suspension with concentrations of OD600 0.2–1.0 for 20 min. The GUS stain (Figure 2E) results showed that the transient expression frequency was increased with the concentration of *Agrobacterium*. The contamination rate of *Agrobacterium* also increased during the subsequent culture (Figure 2C). Therefore, OD600 0.6 was selected as the most suitable infection concentration. In addition, vacuum infiltration was performed to improve the transformation frequency. The transient expression frequency was increased by vacuum infiltration for 5–15 min. However, the calli treated by vacuum infiltration for 15 min showed a higher death rate (50%) in the subsequent selective culture (Figure 2D). It was speculated that a long period of vacuum infiltration may have caused serious damage to the calli. Therefore, soaking for 10 min followed by vacuum infiltration for 10 min was performed for *Agrobacterium* infection.

**FIGURE 3:**
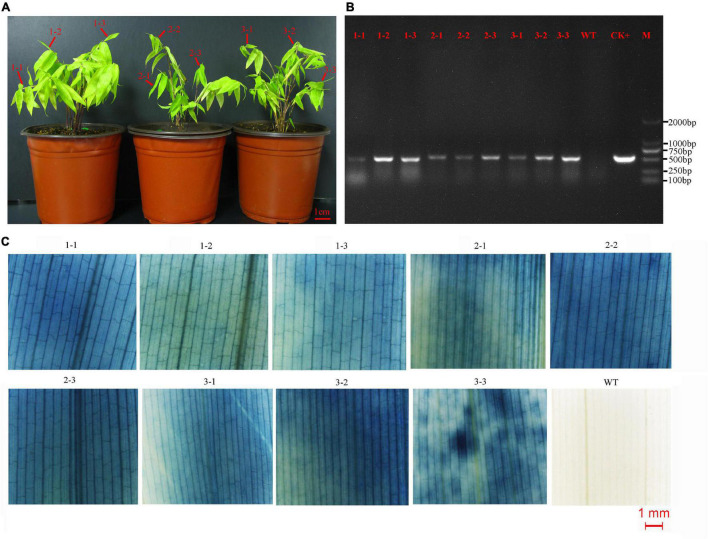
Detection of asexual clones from T0 transgenic lines. **(A)** Three asexual clones of a representative of transgenic line. Leaves of three shoots from each clone were detected by PCR and GUS staining. **(B)** The result of PCR detection. CK+, plasmid; WT, wild type; M, DNA marker (DL2000). **(C)** The result of GUS staining. WT, wild type.

The transformed calli became brown, and new white calli grew from their mother calli after 1.5 months (Figure 2F). The resistant calli were successively subcultured for 3–4 months and recovered vigor for shoot differentiation. Finally, about 23 independent hygromycin-resistant plants were generated from 341 infected calli, of which 17 were positive by GUS and PCR (73.9%) (Figures 2G–I). The transformation efficiency was approximately 5%. It is important to confirm the transgenic nature of asexual clones from T0 transgenic lines. At least three clones of each transgenic line were tested by PCR and GUS staining. The PCR results were 100% positive and GUS was expressed in all plants (Figure 3). These results indicate that the transgenic nature is stable in the asexual reproduction of transgenic moso bamboo plants.

### *PeU3* Promoter Used for Optimizing the CRISPR/Cas9 Genome-Editing System

**FIGURE 4:**
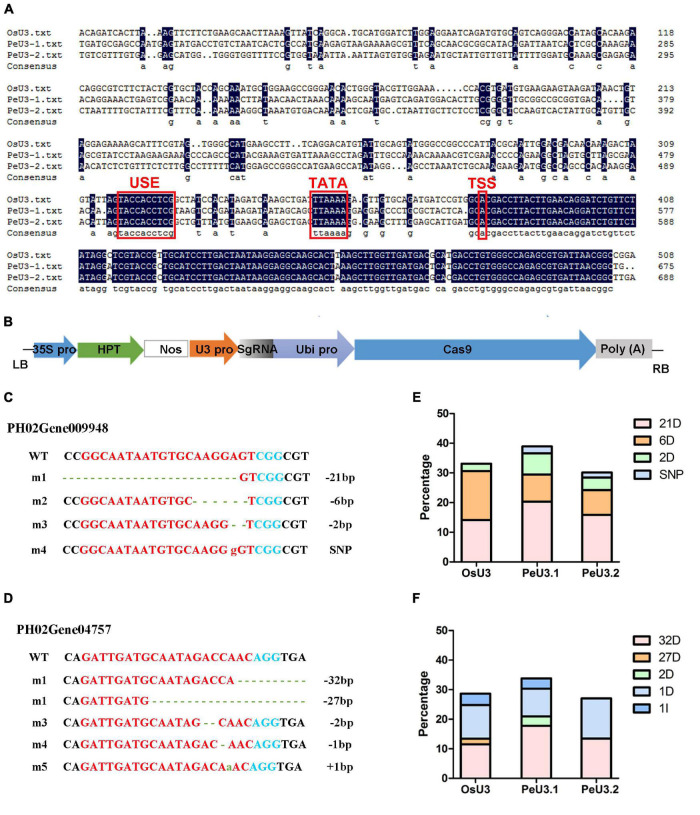
Editing efficiency of two sgRNAs driven by *OsU3/PeU3.1/PeU3.2* promoter. **(A)** Blast result of *PeU3.1*, *PeU3.2*, and *OsU3* promoters. Red box showed USE element, TATA – box and transcriptional start site. **(B)** *pC1300-Ubi:Cas9* vector containing sgRNA site driven by *OsU3/PeU3.1/PeU3.2*, respectively. **(C,D)** Mutation type of sgRNAs targeting PH02Gene09948 and PH02Gene04757. Dotted lines, base deletions; green lowercase letters, base insertions; red lowercase letters, base substitutions. **(E,F)** Frequency of the CRISPR/Cas9-induced mutations in target sites driven by *OsU3/PeU3.1/PeU3.2*, respectively.

To optimize the genome-editing efficiency in moso bamboo, two endogenous U3 snRNA promoters for driving sgRNA expression were identified. Similar to the *OsU3* promoter, the conserved USE element and TATA-box were found in these two *PeU3* promoters (Figure 4A). SgRNA sites targeting PH02Gene09948 and PH02Gene04757 were constructed under the *OsU3/PeU3.1/PeU3.2* promoter and introduced to the expression vector *pC1300-Ubi:Cas9* (Figure 4B). These six constructs were transformed into the calli of moso bamboo following the transformation methods described above. The DNA of 283 resistant calli screened for 1.5 months was extracted, and 198 positive samples (70%) were confirmed by PCR. Thirty samples per *Ubi:Cas9-U3:sgRNA* construct were sequenced using the Hi-TOM protocol (Liu et al., 2019). The results showed that the *PeU3.1* and *PeU3.2* promoters worked and also the *OsU3* promoter. The *PeU3.1* promoter showed higher efficiency (35–39%) when used to drive the gRNA expression for genome editing in moso bamboo (Figures 4E,F). The mutation type was mainly base deletion (Figures 4C,D).

### Homozygous *pds* Mutants Were Obtained by CRISPR/Cas9 Genome Editing

**FIGURE 5:**
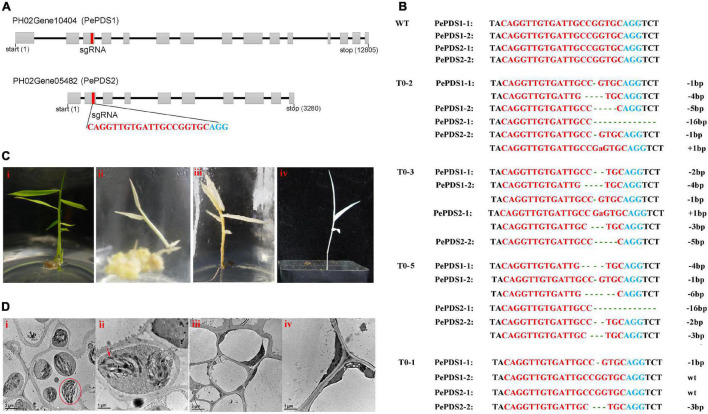
Genome editing of *PePDS* gene by CRISPR/Cas9. **(A)** Genome structures of *PePDS1* and *PePDS2*. Gray boxes, exons; black line, intron; red rectangle, sgRNA target site; red letters, sgRNA target regions; blue letters, PAM regions. **(B)** Mutations at sgRNA site of putative homozygous *pds1pds2* lines (T0-2, T0-3, and T0-5) and a representative of heterozygous mutant (T0-1). Green lowercase letters, base insertions; dotted lines, base deletions. **(C)** Albino shoots and regenerated plant of homozygous *pds1pds2* mutant **(ii–iv)**. **i**, wild type. **(D)** Transmission electron microscope observation of leaves. **i** and **ii**, wild type. **iii** and **iv**, albino mutant. Red circle, chloroplasts; red arrow, thylakoid lamellae.

Two homologous sequences of *OsPDS* were identified, named *PePDS1* and *PePDS2* (PH02Gene05482 and PH02Gene10404, respectively). The further amplified results showed that there were four copies on the moso bamboo genome. One conserved sgRNA site that targeted all copies was designed (Figure 5A). Finally, seven PCR positive lines (70%) were obtained by *Agrobacterium*-mediated transformation. Sequencing results confirmed that six lines (85.7%) were mutated at the sgRNA site. Three lines (42.9%; T0-2, T0-3, and T0-5) were putative homozygous *pds1pds2* mutants (Figure 5B), which exhibited albino phenotypes (Figure 5C) and died during the growth period. However, no visible phenotypic change was observed in heterozygous mutants (T0-1, T0-4, and T0-6), which were mutated at sgRNA site of one or two copies (Figure 5B). Transmission electron microscopy revealed that the green leaves had intact chloroplasts, the thylakoids were arranged in an orderly manner, and the grana lamellae were clear (Figures 5D-i,ii), whereas there were few chloroplasts in the albino mutants and no stacking of thylakoids (Figures 5D-iii,iv).

## Discussion

Genetic engineering technology has been successfully applied to many plant species, but the lack of efficient regeneration system is the major bottleneck of its application in bamboo. There are many factors affecting the regeneration efficiency, among which genotype is the key factor. Because bamboo is mainly propagated by asexual reproduction, there are few bamboo varieties. At present, plant regeneration systems for bamboo have mostly been reported in sympodial bamboo species. The regeneration of grasses is more difficult than that of dicotyledonous plants due to the restriction of explants. Mature or immature embryos are commonly used for plant regeneration in grasses. There have been several reports on plant regeneration from zygotic embryos, such as *B. arundinacea*, *D. latiflorus*, *D. strictus*, and *Otatea acuminata* aztecorum (Mehta et al., 1982; Rao et al., 1985; Yeh and Chang, 1987; Woods et al., 1992). Only one case of a monopodial bamboo species showing plant regeneration has been reported to date. Plant regeneration from calli was induced from the mature seeds of *P. edulis* (Yuan et al., 2013), but the mature seeds were difficult to completely sterilize and no more than 5.0% of the calli were able to regenerate shoots.

Immature embryos are easier to sterilize, and calli can be induced from the scutellum with parenchyma cells. Regeneration systems from immature embryos have been widely established in rice, wheat, corn, and other gramineous crops (Özgen et al., 1998; Frame et al., 2011; Shimizu-Sato et al., 2020). In this study, although bamboo rarely blooms, immature seeds were collected from experienced bamboo farmers every year. The immature embryos were isolated and cultivated as explants to induce calli. It should be noted that even the seeds collected from the same bamboo tree did not develop completely synchronously. Embryos were too young nearly transparent without an obvious bud point, whereas embryos with high maturity were difficult to isolate from the hard endosperm and seed coat. These two types of embryos are not suitable for callus induction. Appropriate seeds appear plump, grayish-green, and the size of immature embryo is about 1.3 mm in diameter (Figures 1A,B). In addition to the type of explants, the basic medium, plant hormones, osmotic pressure, additives, pH, culture conditions, and other factors also affect the efficiency of plant regeneration. Through many preliminary experiments, we found that the concentration of 2,4-D mainly affected the callus induction frequency. As an auxin-like plant regulator, 2,4-D is widely used in callus and somatic embryo induction. The germination of embryos was inhibited with the increase of 2,4-D concentration and the highest frequency of callus induction reached around 60% with the addition of 4 mg⋅L^–1^ 2,4-D in the MS medium. It is generally acknowledged that high concentrations of 2,4-D used in the callus induction stage may not be conducive to subsequent differentiation (Zheng and Konzak, 1999). After 3 weeks of induction, calli were subcultured on MS medium with low concentrations of 2, 4-D (0.1–1.0 mg⋅L^–1^). After long-term subculture, the calli could still maintain strong proliferation and differentiation ability.

Some embryogenic calli (5%) could differentiate into plantlets immediately on the proliferation medium. An optimized combination of cytokinins and auxins was selected to improve the shoot differentiation rate. Recently, transcriptome analysis showed that the expression levels of the majority of ABA-related genes were substantially altered during shoot induction in Ma bamboo, and the exogenous application of an optimized ABA concentration improved the shoot regeneration efficiency (Tu et al., 2021). Our results showed that the shoot differentiation frequency was significantly increased with ABA pretreatment in subculture stage. It is speculated that ABA may act as a “signal” in the formation of embryogenic calli, which needs to be further studied. In addition, it has been reported that some developmental regulators such as *BBM*, *WUS*, and *GRF-GIF* chimeras can accelerate shoot formation in both monocotyledons and dicotyledons (Lowe et al., 2018; Debernardi et al., 2020; Zhang et al., 2021). Next, we will study whether these regulators can further improve shoot differentiation in moso bamboo.

Obtaining stable transgenic plants through *Agrobacterium* mediated transformation is the main means of genetic transformation. Numerous factors, such as the plants genotype, types, and stages of the tissues infected, the strains of *A. tumefaciens*, the concentration of the inoculum, the type of vectors used, the tissue culture media, the selection markers, and the selective agents are of critical importance for achieving efficient transformation. As in rice and Ma bamboo, hygromycin phosphotransferase (HPT) was an efficient selection marker for moso bamboo transformation. We further optimized the *Agrobacterium* concentration and added vacuum infiltration for infection, which improved the transient expression efficiency. However, the main obstacle to transformation was that the regeneration ability of hygromycin-resistant calli was largely reduced and needed to recover for a long period before regenerating plants after the transformation of bamboo (Ye et al., 2017). Selection pressure is also a severe abiotic stress. Even the transformed cells expressing a selection marker gene tend to grow less vigorously on the media that contain a selective agent than on non-selective media (Hiei et al., 2014). AS in Ma bamboo, in this study, 8 months were needed for the whole transformation period. This period may be shortened by optimizing the transformation procedure in future research.

The expression of CRISPR in plants is typically achieved with a mixed dual-promoter system, in which the Cas protein is expressed by a Pol II promoter and a guide RNA is expressed by a Pol III promoter such as U6 or U3. The editing efficiency varies with the use of different U3/U6 promoters for driving sgRNA transcription. Ye et al. (2020) who compared the editing efficiency of three rice U6 promoters (*OsU6a/OsU6b/OsU6c*) using Ma bamboo protoplast system found that *OsU6* showed higher editing efficiency. It is reported that species-specific *U3*/*U6* promoters are much more efficient for driving sgRNA transcription and enhancing the editing efficiency of the CRISRP/Cas9 system (Feng et al., 2013; Sun et al., 2015; Long et al., 2018). For example, the genome-editing efficiency was 1.8–6.3 times higher than that obtained using *AtU6-26* promoter when the sgRNA was driven by native *GmU6-10* promoter in soybean (Sun et al., 2015). To optimize the genome-editing efficiency, two endogenous U3 snRNA promoters were identified from moso bamboo and used to drive sgRNA expression. The results showed that the *PeU3.1* promoter exhibited higher efficiency, and it was used in the subsequent genome editing in moso bamboo.

Phytoene desaturase (PDS) is the primary rate-limiting enzyme that catalyzes the desaturation of colorless phytoene into ζ-carotene, which further converted into lycopene, a colorful compound in the carotenoid biosynthesis pathway (Bai et al., 2016). The deformation or knockout of *PDS* genes affects photosynthesis and carotenoid biosynthesis, which results in albinism and retarded plant growth (Tian, 2015). Therefore, the *PDS* gene has been used as a marker to detect the genome-editing efficiency in several plant species, such as *A. thaliana*, *O. sativa*, *Nicotiana tabacum*, and *P. davidiana* × *P. bolleana*, and so on (Feng et al., 2013; Baltes et al., 2014; Banakar et al., 2020; Wang et al., 2020). Three different PDS gene sequences, including two haplotypes of *PdbPDS1* and one haplotype of *PdbPDS2*, were identified in *P. davidiana* × *P. bolleana.* The results showed that if more than two alleles of the two genes were edited, the transgenic plants appeared albino (Wang et al., 2020). Moso bamboo is considered to be an allotetraploid with high genotype heterozygosity (Guo et al., 2019; Zhao et al., 2021). For genome editing, the homologous and subgenome sequences should be considered to designate precise target sites. In this study, two homologous sequences, *PePDS1* and *PePDS2*, were identified. The further amplified results showed that there were four copies of these two homologous sequences. Therefore, we designed one conserved gRNA site that targeted all copies. Finally, three transgenic lines of putative homozygous *pds1pds2* mutants in the sgRNA site were obtained, which showed albino phenotypes. A precise and efficient CRISPR/Cas9-based gene-editing system was demonstrated for the first time in moso bamboo.

In summary, this study established the first reported immature embryo plant regeneration system and genetic transformation system in *P. edulis*, the most important monopodial bamboo species. The *PeU3.1* snRNA promoter was used to drive sgRNA expression, and an efficient CRISPR/Cas9-based genome editing was demonstrated in moso bamboo. These technical systems will be conducive to gene functional validation and accelerate the breeding process of moso bamboo.

## Data Availability Statement

The original contributions presented in the study are included in the article/Supplementary Material, further inquiries can be directed to the corresponding author/s.

## Author Contributions

GQ and RZ conceived this project, designed the experiments, and interpreted the results. BH, HF, and KJ performed the experiments and analyzed the data. JX and YW helped to perform the experiments and collected the data. BH and GQ wrote the manuscript. All authors read and approved the submission of this manuscript.

## Conflict of Interest

The authors declare that the research was conducted in the absence of any commercial or financial relationships that could be construed as a potential conflict of interest.

## Publisher’s Note

All claims expressed in this article are solely those of the authors and do not necessarily represent those of their affiliated organizations, or those of the publisher, the editors and the reviewers. Any product that may be evaluated in this article, or claim that may be made by its manufacturer, is not guaranteed or endorsed by the publisher.

## Footnotes

1
http://www.bamboogdb.org/#/

## Supplementary Material

Supplementary Video 1 Isolation of immature embryos from the seeds of moso bamboo.
