# Supplementary material for: An Efficient Genetic Transformation and CRISPR/Cas9-Based Genome Editing System for Moso Bamboo (Phyllostachys edulis)
Source: Front Plant Sci. 2022 Feb 11;13:822022. doi: 10.3389/fpls.2022.822022 (PMC8874139; doi:10.3389/fpls.2022.822022)
Supplement: Supplementary file 1 [file Data_Sheet_1.PDF]

Supplementary Material

Genetic transformation protocol of moso bamboo

1. Medium

Callus induction medium (CIM): MS + 4 mg Ł$^{-1}$ 2,4-D + 30 g Ł$^{-1}$ sucrose + 7g Ł$^{-1}$ agar, PH5.8

Callus subculture medium (CSM): MS + 0.1-1.0 mg Ł$^{-1}$ 2,4-D + 30 g Ł$^{-1}$ sucrose + 7 Ł$^{-1}$ agar, PH5.8

***Agrobacterium* infection medium (AIM):** MS + 0.5 mg Ł$^{-1}$ 2,4-D + 30 g Ł$^{-1}$ sucrose, PH5.8

Co-culture medium (CCM): MS + 0.5 mg Ł$^{-1}$ 2,4-D + 0.1mmol Ł$^{-1}$ acetosyringone (AS) + 30 g Ł$^{-1}$ sucrose + 7 Ł$^{-1}$ agar, PH5.8

Selected medium (SM): MS + 0.5 mg Ł$^{-1}$ 2,4-D + 40mg Ł$^{-1}$ hygromycin(Hyg) + 300 mg Ł$^{-1}$ cefotaxime (Cef)+ 30 g Ł$^{-1}$ sucrose + 7 Ł$^{-1}$ agar, PH5.8

Pre-treatment for shoot induction medium (PSIM): MS + 0.5 mg Ł$^{-1}$ 2,4-D + 0.5 mg Ł$^{-1}$ ABA + 40mg Ł$^{-1}$ hygromycin + 300 mg Ł$^{-1}$ cefotaxime + 30 g Ł$^{-1}$ sucrose + agar 7g Ł$^{-1}$, PH5.8

Shoot induction medium (SIM): MS + 0.5 mg Ł$^{-1}$ NAA + 2.0 mg Ł$^{-1}$ BAP + 3.0 mg Ł$^{-1}$ ZT + 25 mg Ł$^{-1}$ hygromycin + 300 mg Ł$^{-1}$ cefotaxime + 30 g Ł$^{-1}$ sucrose + 7 Ł$^{-1}$ agar, PH5.8

Root induction medium (RIM): 1/2MS + 2.0 mg Ł$^{-1}$ IBA + 300 mg Ł$^{-1}$ cefotaxime + 30 g Ł$^{-1}$ sucrose + 7 Ł$^{-1}$ agar, PH5.8

Plant growth medium (PGM): MS + 30 g Ł$^{-1}$ sucrose + 7g Ł$^{-1}$ agar, PH5.8

All media were sterilized at 121 ℃ for 15min. AS, Cef and Hyg were sterilized with 0.22 μm filter.

2. Protocol

(1) Callus induction: the immature seeds were washed under tap water for 2 h and then soaked in 75% ethanol for 1 min. After being sterilized in a 2% sodium hypochlorite solution containing 0.1% Tween-80 for 15–20 min and rinsed with autoclaved distilled water 3–5 times, the embryos were

extruded from the base of the seeds with a sickle probe on the sterile filter paper and placed onto individual 9–cm diameter petri dish containing 20–ml CIM.

(2) Callus subculture: after 3 weeks of induction, light yellow calli were subcultured on CSM (1.0 mg L^{-1} 2,4-D) once every month, and then the white calli were subcultured on CSM (0.1 or 0.5 mg L^{-1} 2,4-D).

(3) *Agrobacterium* infection: the construct was transformed into *Agrobacterium* strain EHA105. Single transformant was cultured on LB solid medium supplemented with kanamycin (50mg L^{-1} and rifampicin 25mg L^{-1}) for 3 days, the bacteria were collected and resuspended with AIM to OD600 0.6. The calli were soaked in the bacteria solution for 10 min followed by vacuum infiltration for 10 min. The infected calli were dried on sterile filter paper for 60 min.

(4) Co-culture: the infected calli were transferred on CCM for 3 days in dark.

(5) Resistant calli selection: after co-culture, the calli were subcultured on the SM once every month in dark.

(6) Pre-treatment for shoot induction: after 3–4 months of selecting culture, the resistant calli were transferred to PSIM for one month in dark.

(7) Shoot induction: the resistant calli were transferred to SIM for 2–3 months in 16h light/8h dark photoperiod. The medium was changed every month.

(8) Root induction and transplanting: the differentiated shoots with 2–3 cm were transferred to RIM for three weeks, and then transferred to PGM for one month. The regenerated plants with 5–7 cm height were transplanted into peat soil and grown in growth chamber at 26 ℃ for 16 h light/8h dark photoperiod.

Culture condition: the calli were induced and proliferated in dark, whereas shoots differentiation and rooting were conducted at 26 ℃ under 16 h light/8h dark photoperiod with the light intensity of 60–70 μmol/m^2/s.
